# A Radio Frequency Region-of-Interest Convolutional Neural Network for Wideband Spectrum Sensing

**DOI:** 10.3390/s23146480

**Published:** 2023-07-18

**Authors:** Adam Olesiński, Zbigniew Piotrowski

**Affiliations:** Communications Systems, Faculty of Electronics, Military University of Technology, 00-908 Warsaw, Poland; zbigniew.piotrowski@wat.edu.pl

**Keywords:** radio frequency machine learning (RFML), cognitive radio, signals detection, object detection using CNN, deep learning

## Abstract

Wideband spectrum sensing plays a crucial role in various wireless communication applications. Traditional methods, such as energy detection with thresholding, have limitations like detecting signals with low signal-to-noise ratio (SNR). This article proposes a novel deep learning-based approach for RF signal detection in the wideband spectrum. The objective is to accurately estimate the noise distribution in a wideband radio spectrogram and improve the detection performance by substracting it. The proposed method utilizes convolutional neural networks to analyze radio spectrograms. Model evaluation demonstrates that the RFROI-CNN approach outperforms the traditional energy detection with thresholding method by achieving significantly better detection results, even up to 6 dB, and expanding the capabilities of wideband spectrum sensing systems. The proposed approach, with its precise estimation of noise distribution and consideration of neighboring signal power values, proves to be a promising solution for RF signal detection.

## 1. Introduction

The detection of RF (radio frequency) signals in the broadband spectrum is of particular importance in cognitive radio solutions, radio frequency spectrum management, signal intelligence, electronic warfare or new-generation telecommunication network solutions. It is therefore an essential part of radio frequency spectrum monitoring, and monitoring is one of the steps in building awareness of the electromagnetic situation. Consequently, the performance of the aforementioned systems depends on correctly designed detectors, their parameters, decision-making methodology and detection speed.

The radio signal is actually a variable electrical signal induced by electromagnetic waves at the antenna terminals. In modern solutions, it travels through a waveguide to a receiver based on the SDR (software-defined radio) architecture. SDR technology is defined by IEEE 1900.1 [1] as a radio for which some or all of the physical layer functions are software-defined. However, the radio signal from the antenna is too weak to be sampled directly, so it is amplified by the LNA (low noise amplifier). Very often, there is also an input filter assembly before the LNA module, which reduces the bandwidth of the signal being amplified to reduce the potential for overdriving the amplifier, and thus reduce the likelihood of intermodulation. The amplified RF signal then goes to a frequency conversion circuit and an analog-to-digital converter (superheterodyne or direct conversion solutions) or directly to an analog-to-digital converter (direct sampling solutions). Other stages of radio signal processing are implemented through software, e.g., in FPGAs (field-programmable gate arrays), DSPs (digital signal processors) or a PCs (personal computers).

An undeniable advantage of SDR is its reconfigurability, i.e., changing the received and transmitted waveform. Taking additionally into account the growing interest in cognitive and intelligent radio solutions, it has become a natural evolution of cognitive radio to apply machine learning techniques to the radio environment. RFML (radio frequency machine learning) systems have been described by DARPA [2] as performing four basic tasks, namely RF fingerprinting, RF fingerprint enhancement (which can be realized by using radio-based steganographic solutions [3,4] in DNNs), spectrum awareness and autonomous RF system configuration. Detecting signals in the broadband frequency spectrum fits directly into spectrum awareness, and using convolutional neural networks, or deep learning, as a detector fits into the broader conceptual set of machine learning.

In the literature, we can find applications of deep neural networks, among others, in the classification of radio signals [5,6] or the use of DNNs as a detector in the basic processing band in the detection of signals with known characteristics [7,8]. However, there are very few approaches for the detection of signals in a wideband spectrum, returning proposals of regions of interest, i.e., center frequency and occupied bandwidth; and time stamps of occurrence of the signal of interest in the radio electromagnetic environment, i.e., de facto RPNs (region proposal network) for radio signals.

The article introduces a novel deep learning-based approach for detecting RF signals in wideband spectrum, accurately estimating the noise distribution in a wideband radio spectrogram. It covers the topic of wideband spectrum sensing, explores object detectors that employ deep neural networks, introduces the dataset utilized for the research and proposes the architecture of RFROI-CNN. Additionally, the model undergoes evaluation, and the findings are compared to those of a detector based on energy detection with thresholding.

The application of convolutional neural networks for the analysis of radio spectrograms yields good results, as the estimation of noise distribution is performed through convolutions with a kernel size of 3 × 3, thus taking into account neighboring signal power values in the analyzed spectrogram segment. The proposed approach proves to be significantly better than the commonly used energy detection method with thresholding, up to 6 dB, allowing for the detection of signals in a wideband spectrum with low SNR values.

## 2. Wideband Spectrum Sensing

Various signal detection methods are used in radiocommunications, surveying, radar, sonar or other signal processing systems. These include the energy detector [9,10], matched filter [11], correlation detector [12], cyclostationarity detector [13], wavelet detector [14] or covariance detector [15]. In the case of devices or systems analyzing the radio frequency spectrum, such as cognitive radio, the correct detection of radio signals in the electromagnetic spectrum is a key issue defining the usability of a given solution in a specific operational scenario.

Radio spectrum sensing methods can be divided into narrowband, i.e., those in which the frequency response of the communication channel is considered as flat; and broadband [16]. Narrowband methods allow for high dynamic detection with relatively low computational complexity; however, broad-frequency spectrum monitoring is not possible. In contrast, broadband methods tend to have much lower detection dynamics than narrowband methods, but they allow for multiple radio channels to be monitored concurrently.

In general, we can state that if the signal received in band B is only additive noise the band is considered to be free (hypothesis $H_{0}$), on the other hand, when the signal received in this band additionally contains a transmission signal, it is considered to be occupied (hypothesis $H_{1}$). Therefore, the role of each RF detector is to choose one of the two hypotheses ($H_{0}$ or $H_{1}$), described by Equations (1) and (2).

(1) $$H_{0}:y\left(n\right)=w\left(n\right)$$

(2) $$H_{1}:y\left(n\right)=s\left(n\right)+w\left(n\right)$$

where y(n) is a signal at the detector input, s(n) is useful signal in the channel, w(n) is noise, n=0,1,2,3,…,N—consecutive signal samples. As a qualitative measure of the detector, the most commonly used are the probability of correct detection $P_{d}$, which is the probability of selecting hypothesis $H_{1}$ when it is true (true positive); and the probability of false detection $P_{f}$, which is the probability of selecting hypothesis $H_{1}$ when it is false (false positive). Therefore, a well-designed RF detector maximizes $P_{d}$ while minimizing $P_{f}$.

Typically, measurement equipment, broadband receivers dedicated to spectrum sensing and analysis, or cognitive radio solutions use energy detection (ED) [17] or its expansions (e.g., ED-ENP [16,18]) as a method to detect signals in the radio spectrum. This is due to the fact that in this method, there is no need to have information about the characteristics of the signals present in the spectrum, which is crucial for the versatility of the systems and their operation in any state of the radio environment and for maintaining basic compatibility with newer waveforms. In addition, appropriate preprocessing is often used to increase processing gain, such as adaptive filtering or spectrum averaging. This allows the noise floor to be lowered, which increases $P_{d}$ for continuous signals with low SNR (signal-to-noise ratio), but removes the ability to record pulsed signals with low SNR.

For an ideal energy detector, we can write the statistical test equation for a signal y(n) with number of samples *N* and noise power $\sigma^{2}$ [16]:

(3) $$\Lambda \left(y\right)=\frac{1}{2\sigma^{2}}\frac{1}{N}\sum_{n=1}^{N}{\left|y\left(n\right)\right|}^{2}\begin{array}{c} H_{1}\\ \geq \\ <\\ H_{0} \end{array}\lambda$$

As typically in real spectrum sensing systems, the noise variance is unknown, ED-ENP (estimated noise power energy detector) detectors [16] are used, estimating the noise power ${\hat{\sigma }}^{2}$, usually using Equation [16]:

(4) $${\hat{\sigma }}^{2}=\frac{1}{2M}\sum_{n=1}^{M}{\left|w\left(n\right)\right|}^{2}$$

where w(n)—noise, n=0,1,2,3,…,M—consecutive noise samples. Therefore, a test can be written for the ED-ENP detector [16]:

(5) $$\Lambda \left(y\right)=\frac{1}{\sum_{n=1}^{M}{\left|w\left(n\right)\right|}^{2}}\frac{M}{N}\sum_{n=1}^{N}{\left|y\left(n\right)\right|}^{2}\begin{array}{c} H_{1}\\ \geq \\ <\\ H_{0} \end{array}\lambda$$

For most detection methods, at one stage of signal processing, it is necessary to define a decision threshold λ deciding on the hypothesis $H_{0}$ or $H_{1}$ for the signal y(n). Often, the threshold value is variable and determined by the current electromagnetic situation. There are also statistical solutions to keep the probability of a false alarm low, such as the Neyman–Pearson criterion or other more or less complex adaptive methods [17].

## 3. Object Detectors Based on Deep Neural Networks

Deep (including convolutional) neural networks have proven their effectiveness in classification [19], similar data search [20], compression [21], denoising [22,23] or new image generation [24]. They are also used in object detection in images or video, among other applications. Typical deep neural network-based object detectors are, in fact, suitably modified classifiers. Classification involves assigning a label to the input data. For images, the best classification results are obtained when the object fills most of the image and is centered [25]. For real data such as photos or images from surveillance cameras, we are dealing with more complex content, i.e., the presence of multiple objects of different sizes. Because of that, a standard classifier will not provide a suitable description of the input data. Object detectors based on convolutional neural networks are therefore a natural evolution, allowing not only for the assignment of multiple labels to input data, but also for the indication of the area in space where the object associated with a particular label is located.

CNN detectors can be divided into two categories: two-stage [26,27,28,29,30] and single- stage [31,32,33,34,35]. In the case of two-stage detectors, the first stage consists of proposing regions in which objects may be located. Typically, selective search algorithms [36], EdgeBoxes [37] or other algorithms, including region proposal networks based on, e.g., CNN, are used for this. The next stage is to carry out classification on each of the proposed regions. Examples of two-stage detectors include R-CNN [38], Mask R-CNN [26], Fast R-CNN [27], Faster R-CNN [28], FPN [29] or relation networks [30]. While two-stage detectors typically have higher accuracy than single-stage models [25], in terms of performance, the first models were too slow to be used in real-time solutions. Newer solutions like the Faster R-CNN VGG-16 can achieve 7FPS when used with the PASCAL VOC 2007 database [39].

Single-stage detectors lack the region proposal stage—all objects are detected during a single pass of data through the network. Examples of one-step detectors include YOLO [31], YOLO9000 [32], YOLOv3 [33], SSD [34] and RetinaNet [35]. The biggest advantage of single-stage models is their speed with satisfactory accuracy [40].

Neural networks that include processed data in their structure or work on Fourier transform [41,42,43,44] or wavelet transform [45,46,47] are interesting. They enable the extraction of features hidden in signals. Most often, in the case of deep neural networks, sound, such as music or human speech, is subjected to the Fourier transform so that classification is performed on PSD histograms [48,49,50]. A similar approach can be applied to the radio environment, i.e., to analyze the radio frequency spectrum of the electromagnetic environment. In radio communications, the Fourier transform of a signal is a common block in the signal processing chain, enabling frequency analysis of the signal, filtering or subjecting signals to secondary modulation such as OFDM [51]. There are publications using FFT in the RF environment and demonstrating the use of convolutional detectors in processing RF spectrograms, with positive results [8].

So far, in wideband spectrum analysis, technical methods based on energy detection with thresholding have been commonly used. However, attempts to apply popular computer vision convolutional detectors on radio spectrograms have yielded mediocre results. This is because convolutional detectors operate as classifiers, and classifying radio signals in spectrograms for different SNR values, modulations, observation bandwidths, interferences, etc., leads to many false detections. It should be noted that no publication has been found for a CNN-based universal radio detector that does not operate as an object classifier, like RFROI-CNN.

## 4. RFROI-CNN Proposal

Typically, convolutional neural networks achieve high levels of correct classification and detection on data with a feature distribution similar to the data on which the network training process took place. For example, a network trained on images with human silhouettes captured at close range with a high-resolution camera may struggle to achieve high-quality classification rates on images from a low-resolution CCTV camera where human silhouettes are in the distance. In addition, changing the size of the image without preserving the proportions, and thus changing the distance between the characteristics of the human figure, also negatively affects the accuracy of the classification.

The same problem is even more pronounced for the classification and detection of signals in the radio spectrogram. In real conditions, the radio spectrum contains signals of high and low energy—different SNR values, different primary and secondary modulations, or bandwidths occupied in the spectrum. The time and frequency resolution of the Fourier transform also affect the feature distribution. Moreover, in spectrum sensing applications, when we do not expect a specific transmission with known characteristics, it is difficult to have a good enough training database containing most of the waveforms occurring in the real radio environment, i.e., primary and secondary modulations, to be able to accurately classify and detect all the signals occurring in the spectrum with conventional CNN-based solutions.

Therefore, this paper proposes a different approach to the detection of RF signals in a broadband spectrogram than standard CNN-based object detectors [26,27,31]. By analyzing Equation (2), we can see that the probability of detection in the energy detection method can be increased by maximizing the SNR, i.e., either increasing the signal energy in the channel or decreasing the noise energy. Knowing the good results of using CNNs in image denoising [22,23] and restoration of masked parts of images [52,53,54], a universal RF signal detector based on CNN estimating noise was implemented. In this case, radio signals are treated as masks that obscure the actual noise distribution. The solution we present below is *de facto* a broadband convolutional ED-ENP detector, where instead of a channel noise power estimate, the noise power distribution in the spectrogram is estimated.

### 4.1. Radio Signals Database

**Figure 1:**
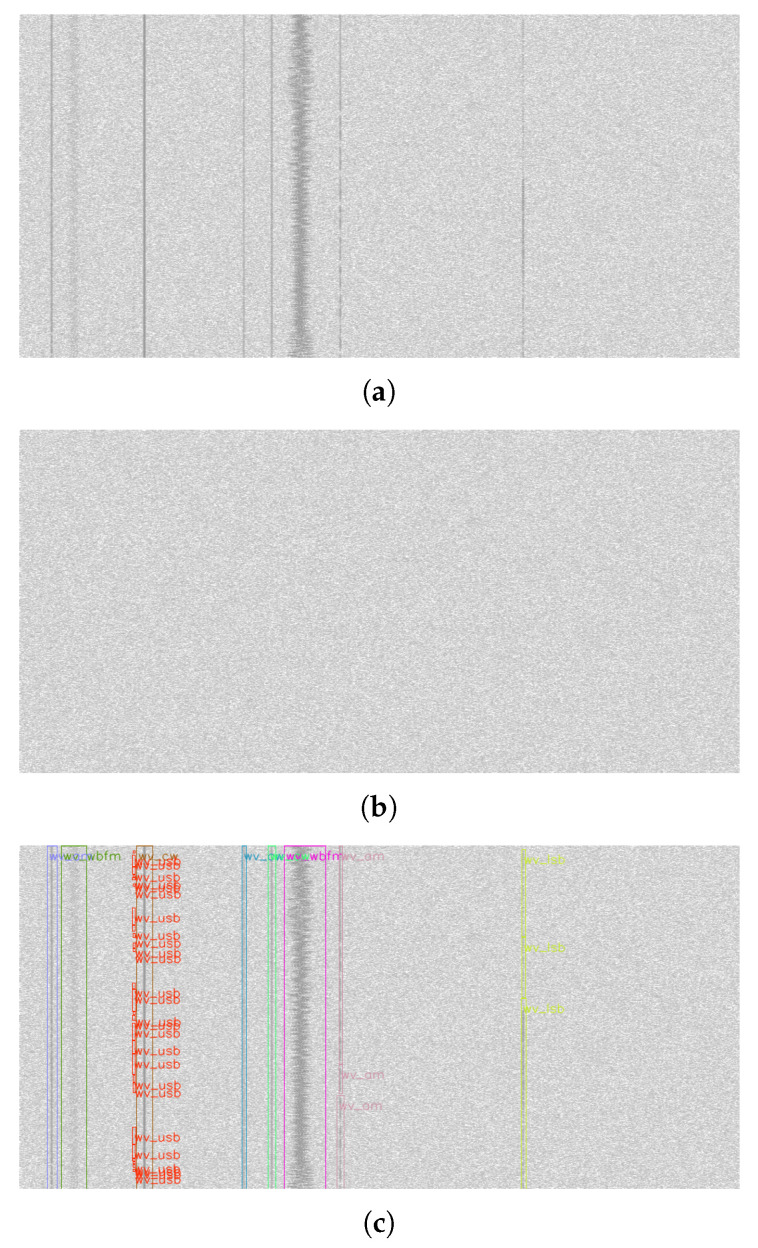
Example of wideband radio spectrogram (**a**) and noise distribution (**b**) pair from the rfspec-db database. In addition, visualized bounding boxes of existing signals (**c**) are shown.

There are databases of radio signals that can be used in RFML [55,56,57,58], but there are no publicly available databases that meet the requirements of capturing radio signals not in the baseband but as part of a broader radio spectrum snapshot. Of course, it is possible to take actual snapshots of the radio environment, but this deprives of the ability to accurately distinguish signal from noise, not to mention having accurate information about the time domain of noise without signals, which is required in training the network for the noise estimator task. This creates a need for the generation of this type of database: a solution was created to generate a synthetic database of radio signals in the broadband spectrum. The task of the software is to generate synthetic radio spectrograms from randomly generated spectrum recipes, i.e., to extract data from the recipe regarding the sampling frequency; the duration of the spectrum snapshot; the waveforms used; the frequencies at which the resulting waveform signals are to be placed; and the SNR—or in fact, because the network is trained on radio spectrograms, the PSNR (peak signal-to- (average) noise ratio):

(6) $${PSNR}_{dB}=10log_{10}{\left(\frac{max(|S_{n}|)}{A}\right)}^{2}$$

where $S_{n}$ is the signal sample vector and *A* is the average noise amplitude. The software uses GNU radio to easily generate radio waveforms. The composite IQ samples of the waveforms are generated in the time domain and then transferred into the RF domain—i.e., into a given sampling rate and carrier frequency by an interpolator, digital upconverter and bandpass filter—and stripped of the complex component. They are then all summed together with their respective weights (PSNR) and AWGN (additive white Gaussian noise) channel noise. The time domain channel noise at the appropriate sampling rate is captured separately. The project was named rfspec-db-synthesizer. For network training, a database rfspec-db(M(AM; FM; CW; LSB; USB; OFDM), PSNRdB(−4; 12)) was generated, i.e., containing randomly distributed transmissions in spectrograms with AM (amplitude modulation), FM (frequency modulation), CW (continuous wave), LSB (lower sideband modulation), USB (upper sideband modulation) and OFDM modulations with a random PSNR factor ranging from −4 dB to +12 dB. An example database record containing nine radio signals is shown in Figure 1, where Figure 1a is a wideband radio spectrogram with visible radio signals, Figure 1b is a noise power distribution spectrogram and Figure 1a,c is bounding box visualization of signal occurrences. The darker the shade of the spectrogram means the higher the signal power. We provide the rfspec-db database used for training to reproduce the experiment by others [59].

### 4.2. RFROI-CNN Structure Proposal

**Figure 2:**
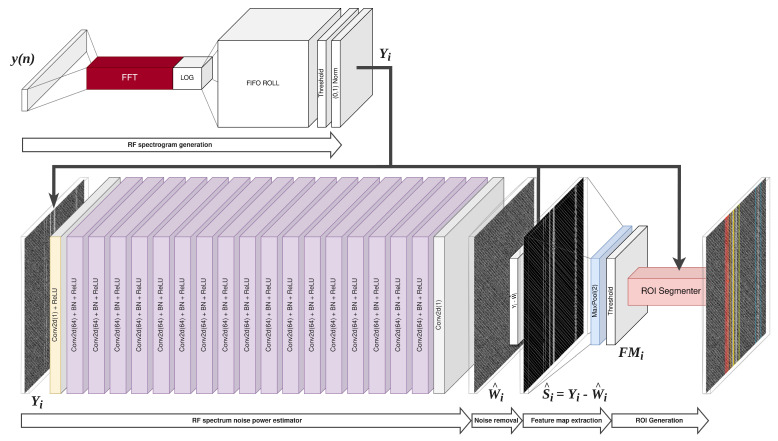
Proposed RFROI-CNN solution.

The proposed solution structure is shown in Figure 2. The main core is a fully convolutional network containing 19 convolutional blocks with a kernel size of 3 × 3, without max pooling layers. The input layer takes one feature, and the result is subjected to the ReLU activation function without batch normalization; the output layer returns one feature without ReLU and without BN; the others have a depth of 64 features, BN and ReLU, similar to the DN-CNN network [23]. The input data from the database, i.e., the signals summed with the channel noise y(n)=s(n)+w(n), and separately the channel noise w(n), are subjected to the Fourier transform and stored in a LIFO queue created in the GPU memory space. The transform result is converted to a decibel measure to highlight weak signals in the spectrum. They are then subjected to thresholding and normalization. The thus-normalized spectrograms of the spectrum Y and the spectral noise W form an indivisible training batch.

A typical loss function was used $L({\hat{W}}_{i})$ for regression and classification solutions—MSE (mean square error) (Equation (7)), where *i* is the batch element index and *N* is the batch size.

(7) $$L\left({\hat{W}}_{i}\right)=\frac{1}{N}\sum_{i=1}^{N}\left|\right|{\hat{W}}_{i}-W_{i}{\left|\right|}^{2}$$

The network is trained to act as an estimator of channel noise ${\hat{W}}_{i}=R\left(Y_{i}\right)\approx W_{i}$, i.e., a spectral image containing signals and noise is given as input, and a loss function is calculated between the network output and the noise spectrogram. With an estimate of the spectral noise, we can extract the signals within it. Due to the logarithmic representation of the spectrum, it seems most sensible to divide and subtract the offset, i.e., ${\hat{S}}_{i}=\frac{Y_{i}}{{\hat{W}}_{i}+k}-1$, where *k* is very little value constant, preventing division by 0. If ${\hat{W}}_{i}=W_{i}$, this method works correctly; however, for some of the estimates, it results in an inability to determine the correct λ-threshold. Therefore, subtraction is applied, i.e., the i-th spectral noise estimate is subtracted from the i-th spectrogram (Equation (8)):

(8) $${\hat{S}}_{i}=Y_{i}-{\hat{W}}_{i}$$

where ${\hat{S}}_{i}$ is the signal estimate. Therefore, we can write the hypotheses previously mentioned in Equations (1) and (2) as:

(9) $$H_{0}:{\hat{S}}_{i,c,h,w}=0$$

(10) $$H_{1}:{\hat{S}}_{i,c,h,w}>0$$

where *c* is the input feature (always 1), and *h* and *w* are the feature indices, respectively, corresponding to the frequency and time axes in the spectrogram, and hence, the power at a given frequency at a given instant. In fact, ${\hat{W}}_{i}\neq W_{i}$; only ${\hat{W}}_{i}\approx W_{i}$, hence the need to further process the estimate ${\hat{S}}_{i}$, which undergoes the MaxPool(2,2) undersampling operation, the result of which is ${\hat{S}}_{i}^{\prime }$; and then thresholding according to Equation (11), where λ is the decision threshold, and for case ${\hat{W}}_{i}=W_{i}\Rightarrow \lambda =0$. We can treat the thresholding result as a binary map of the ${\mathrm{FM}}_{i}$ features, containing the masks of all detected signals in the radio spectrum.

(11) FMi,c,w,h=0 if S^i,c,h,w′≤λ1 if S^i,c,h,w′>λ

A feature map with twice the resolution in both axes goes into ROI segmenter, where bounding box coordinates are extracted based on the continuity of binary features in ${\mathrm{FM}}_{i}$. Built-in OpenCV functions were used, although this can also be carried out through a suitably designed neural network. Of course, appropriate postprocessing is necessary so that the coordinates are converted to the center frequency $f_{k}$ and the bandwidth $B_{k}$ and the timestamp $T_{k}$, where k=0,1,2,…,N—the next signal detected in the spectrum.

## 5. Experiments

### 5.1. Database and Training Settings

**Figure 3:**
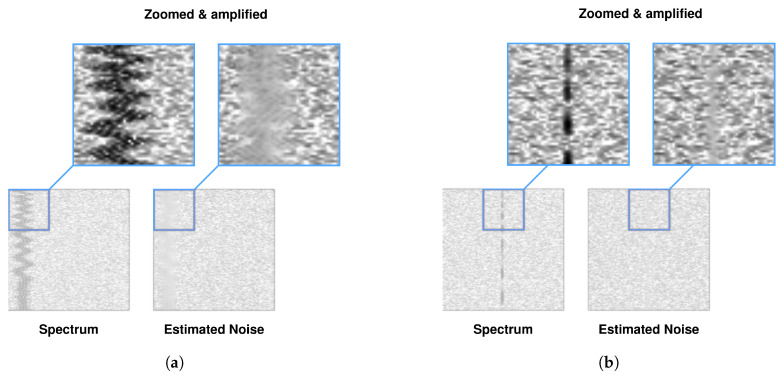
Example spectrograms $S_{i}$ for WBFM (**a**) and SSB (**b**) from rfspec-db database and noise estimates ${\hat{W}}_{i}$ from RFROI-CNN.

The network was trained on a database containing 391 {Y,W} pairs [59]. The following was applied: FFT size ∈{1024,2048,4096,8192}, time resolution $T_{R}\in \left\{256,512,1024,2048\right\}$, sampling rate (in Hz) $f_{s}\in \{$$2\;\times \;10^{6}$$,5\;\times \;10^{6},\;10\;\times \;10^{6},\;15\;\times \;10^{6},\;20\;\times \;10^{6},\;25\;\times \;10^{6},$$30\;\times \;10^{6},\;35\;\times \;10^{6},$ $40\;\times \;10^{6}\}$, durations in seconds t∈{0.25,0.5,0.75,1}, spectrum type—real. These 391 pairs were divided from stride 200 into 200 × 200 patches, resulting in 5189 smaller pairs $\left\{Y^{\prime },W^{\prime }\right\}$. The generated noise in the AWGN channel had a variable randomized seed prior to generation, which counteracted the repetition of the noise distribution, noise amplitude A=0.0025. Patches of spectrograms, using additional annotation files, were checked for the presence of radio signals; if none were present, the patch was discarded. Different proportions of patches with noise alone were also experimented with, while convergence was achieved fastest for patches always containing at least one signal. The thresholding range of the FFT results was empirically selected to be −110 dBm, +5 dBm, with a random augmentation of ±3 dB. In addition, the spectrogram was randomly rotated by ang∈{0,90,180,270} degrees and reflected vertically and horizontally as part of the augmentation. Learning rate $lr=10^{-3}$, batch size $b_{s}=8$. The network model was implemented in PyTorch. The network was trained for 70 epochs on an RTX3060 GPU. Figure 3 shows an example of the noise estimates of the radio spectrogram for the trained network, together with the zoomed and amplified section of the spectrogram and noise estimate.

### 5.2. Results on the Database Test Set

It was problematic to determine the correct measure of detection accuracy using the test set. As mentioned earlier, typically $P_{d}$ and $P_{f}$ are used, but these are measures suitable for classical narrowband detectors. For a broadband detector operating on an RF spectrogram, in addition to the detection itself, the center frequency and bandwidth as well as the start and end of the signal occurrence must be indicated. When generating the database, we have all the information about the signal, i.e., its frequency, modulation, bandwidth, PSNR or timestamps. Therefore, signal masks and corresponding bounding boxes were generated to compare the results. For the detection problem in machine learning, mAP (mean average precision) [60] is most commonly used as a comparison metric; however, due to the intentional lack of classification in this solution, the test database had to be modified accordingly so that one radio spectrogram contains only one signal. The mAP is a precision–recall curve-based metric; hence, it takes into account TP, FP, FN (true positives, false positives, false negatives), and the TP decision is made based on the IoU (intersection over union) threshold [60].

**Figure 4:**
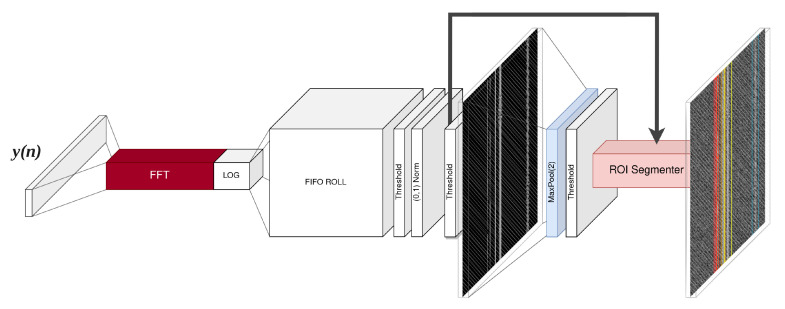
Schematic of the energy detector used for performance comparison.

A dataset rfspec-db-test containing analog frequency and amplitude modulations was prepared and PSNR values were assigned as class labels ∈{−8,−4,−2,0,2,4,6,8} dB. The size of the patch was set to 150 × 150. Instantaneous energy detection by thresholding was used as a comparative detection technique, and thresholds were set λ∈{(0.6;0.96)} with a step of 0.02 of the maximum power in the spectrogram. The value of λ was fixed for each element in the spectrogram fed to the detector input; no adaptive algorithms were implemented. To make the results generated by the energy detection method more reliable, the thresholding module was also attached to the MaxPool(2,2) layer as in the RFROI-CNN scheme, i.e., the spectrogram, after thresholding, was subjected to the same pooling and subsequent thresholding and segmentation operations in ROI segmenter. A schematic of the ED detector is shown in Figure 4.

Due to the specificity of the radio signal detection problem, it was decided to present a typical mAP@.5 measure as well as mAPs with lower IoU threshold values. The energy detector results are shown for the λ, for which the highest mAP value for a given PSNR was obtained. Due to the different specificity of the problem than in the case of detection of objects in photographs, it was decided to prepare test datasets for the difficult case of detection in the broadband spectrum, i.e., analog signals with constant carrier power (AM, FM, CW) and instantaneous power depending on the modulating signal: single−sideband modulations LSB and USB.

**Figure 5:**
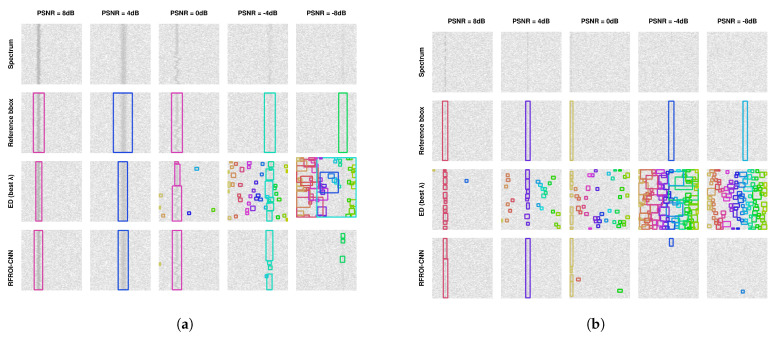
Comparisons of RFROI-CNN and ED results for spectrograms containing WBFM signals (**a**) and SSB signals (**b**) for PSNR values of 8 dB, 4 dB, 0 dB, −4 dB, −8 dB.

**Figure 6:**
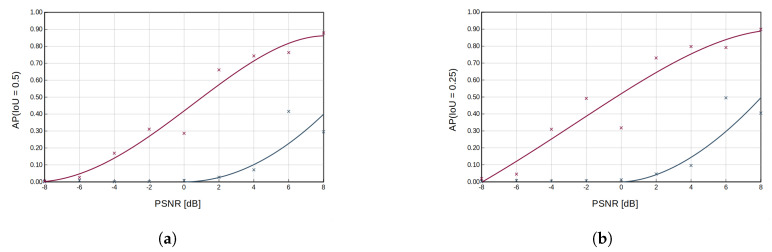

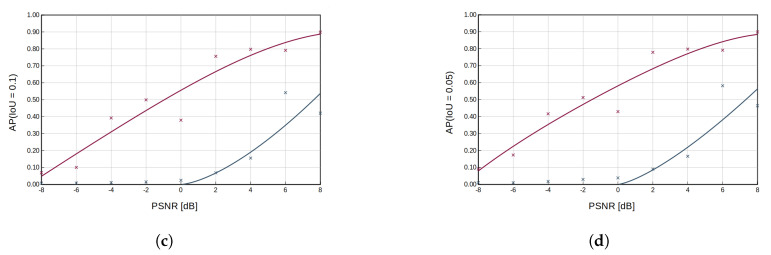
Plot of average precision as a function of PSNR for AM and FM analog modulations and different IoU cut-off thresholds (0.5 in subfigure (**a**), 0.25 in subfigure (**b**), 0.1 in subfigure (**c**), 0.05 in subfigure (**d**)). The magenta color indicates the achieved results for the RFROI-CNN solution; the grey color for the ED energy detector with a threshold λ, for which the highest APs were achieved.

**Figure 7:**
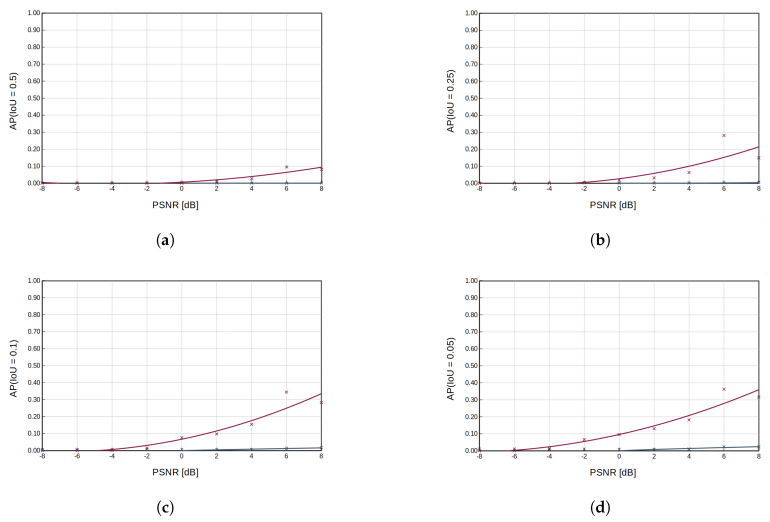
Plot of average precision as a function of PSNR for LSB and USB analog modulations and different IoU cut-off thresholds (0.5 in subfigure (**a**), 0.25 in subfigure (**b**), 0.1 in subfigure (**c**), 0.05 in subfigure (**d**)). The magenta color indicates the achieved results for the RFROI-CNN solution, the grey color for the ED energy detector with a threshold λ, for which the highest APs were achieved.

Figure 5 shows the detection results of bounding boxes for ED and RFROI-CNN. The first row of spectrogram in Figure 5a,b is a radio spectrogram from the test set. The second one is a visualization of a reference bounding box used in mAP computation, created on the parameters of the generated signal. The third row shows graphically plotted signal detection using the ED detector, while the fourth row shows the graphically plotted signal detection using RFROI-CNN. The colors of the bounding boxes are dependent on the center frequency of a detected signal. A summary of the results for the different datasets is shown in Table 1, while the obtained mAP versus PSNR dependencies for IoU threshold ∈{0.5,0.25,0.1,0.05} for fixed carrier power modulations is shown in Figure 6, and for single-band modulations in Figure 7.

Analyzing the results in Table 1 and Figure 6 and Figure 7, it can be seen that RFROI-CNN achieves much higher mAP rates and still works with satisfactory results for PSNRdB < 0. Dataset rfspec-db-test(M(AM; FM; CW), PSNRdB(0; 8)) achieves mAP (IoU = 0.5) = 76.9%, and for rfspec-db-test(M(AM; FM; CW), PSNRdB(−8; 8)) also achieves satisfactory mAP (IoU = 0.5) = 55.27%. For M(AM; FM; CW; LSB; USB), PSNRdB(−8; 8) the difference in mAP (IoU = 0.5) between RFROI-CNN and ED is 20.7%, while for PSNRdB(0; 8), it is 34.31%. For datasets with modulation set M(LSB; USB) for RFROI-CNN and ED, much lower mAP values are obtained, but also in this case, the proposed solution based on convolutional neural networks proves to be better. Additionally, analyzing Figure 5, we can see that in the case of ED and PSNR < 0, apart from the lower TP, the number of false detections increases rapidly, which is not in the RFROI-CNN case.

The problem of the mAP (IoU = 0.5) metric for analog single−band modulations as a function of peak power ratio rather than average-to-noise ratio is that it is not possible to simply synthesize correct signal masks, since the instantaneous power of the modulated signal at the receiver is proportional to the instantaneous power of the modulating signal, as is well demonstrated in Figure 5b. Even at high PSNR, the ED detector is marked by smaller bounding boxes whose horizontal edges mark the moment when the instantaneous SSB signal power drops below the sensitivity threshold, hence the inclusion in the results of mAP (IoU = 0.05), which is a better but still imperfect measure for fading signals, with respect to PSNR. For single-band amplitude modulation (Figure 7), the mAP for the energy detection method is very low—no more than 3% at mAP (IoU = 0.05) (Figure 7d) and PSNR = 8 dB—while for the same conditions, RFROI-CNN performs surprisingly well, as further shown by the results in Figure 5b.

### 5.3. Results in a Real Radio Environment

**Figure 8:**
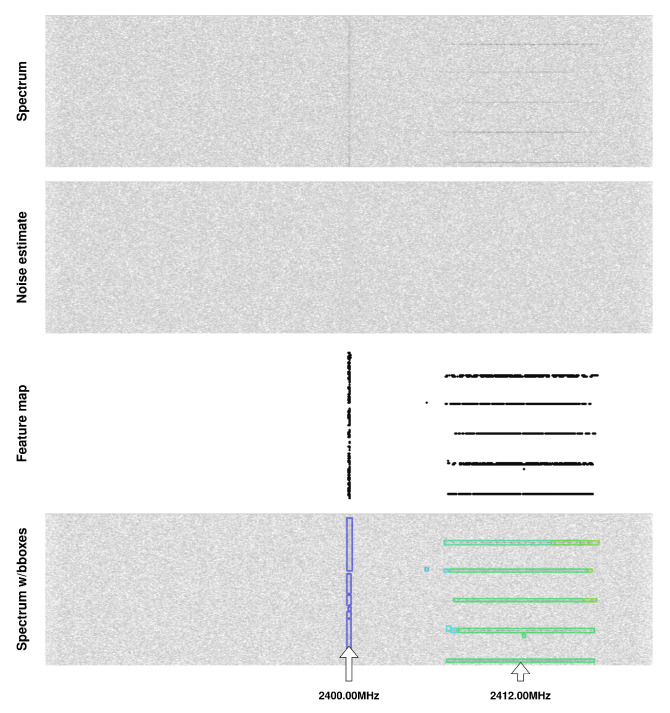
Example signal detections in a real radio environment (band 2400 MHz). From top: radio spectrogram, spectrogram of noise estimate, generated feature map and bounding boxes plotted on the spectrum.

**Figure 9:**
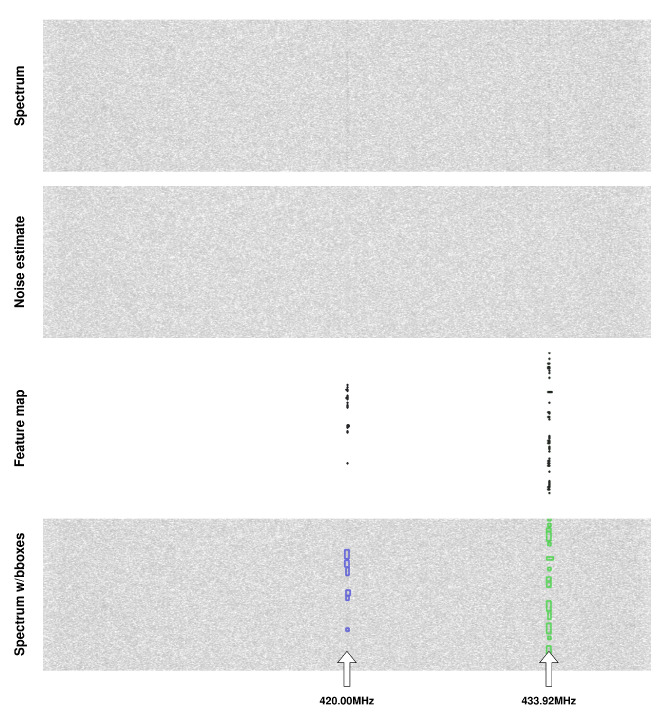
Example signal detections in a real radio environment (band 420 MHz). From top: radio spectrogram, spectrogram of noise estimate, generated feature map and bounding boxes plotted on the spectrum.

The RFROI-CNN solution was developed for use in a real radio environment, and therefore, performance was verified with real-time SDR receivers. The SDR receiver was operated by GNU radio v.3.10.0.0 software, in which a fast Fourier transform and rescaling to logarithmic scale were performed on the sample stream, and the resulting vectors were made available through the ZMQ socket. Data from the ZMQ were queued in GPU memory and then fed to the input of the convolutional network. The resulting detections were plotted on a radio spectrogram without unnecessary delays. Common parameters SR= 42 MHz, λ= 0.05, $G_{LNA}=$ 8 dB were set. Figure 8 and Figure 9 show example detections of RF signals in a real radio electromagnetic environment. In Figure 8, 2.4 GHz band spectrum is shown. The signal visible in the middle of the spectrogram is LO leakage, while the bounding boxes with f= 2412.00 MHz are signals coming from Wi-Fi devices. In Figure 9, the 420 MHz band spectrum is shown. Bounding boxes with *f* = 433.92 MHz are probably a recorded weak signal from a car or gate remote control.

Due to the lack of reference bounding boxes in a dynamic real-world radio environment, it was not possible to calculate mAP.

**Figure 10:**
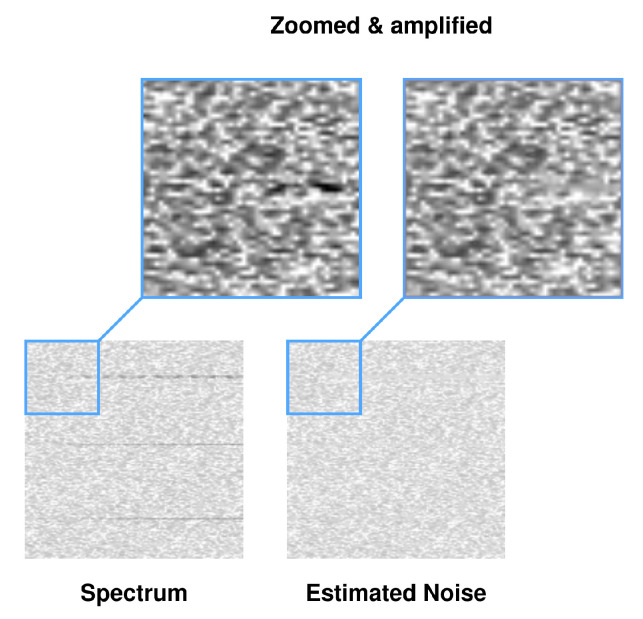
Part of the spectrogram from Figure 8 ($f_{0}$ = 2400.00 MHz) and its estimate of the noise distribution.

Figure 10 shows an excerpt of the zoomed and amplified spectrogram and noise estimate for the case $f_{0}$ = 2400.00 MHz from Figure 8, where the signal normalization performed by CNN, occurring to the mean and std of the noise distribution, is clearly visible.

**Figure 11:**
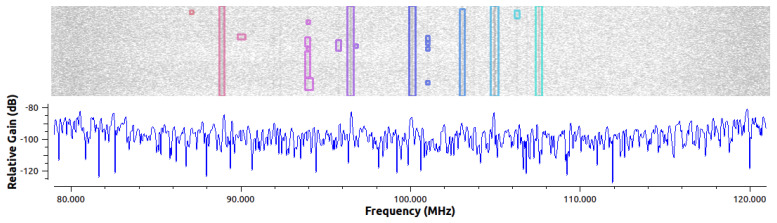
Real broadcast band radio spectrum in the frequency domain (**bottom**) and time–frequency domain (**top**) with the detections from RFROI-CNN plotted.

In addition to correct detection of weak signals, it was expected that the RFROI-CNN solution, with GPU support, could be applied in real time. For the detection of radio signals in the spectrum, the real-time approach is slightly different than, for example, in video images. For object detection in a video stream, we can say that a dozen or so frames per second is a good enough result. For radio signals, however, it depends on the waveform we want to detect with a certain precision. For very fast solutions, e.g., TDD, that use a single frequency, it is still sufficient to observe the signal, extract the center frequency and bandwidth, and then further analyze in the baseband. However, in the case of frequency-hopping radios, which perform, for example, several hundred frequency hops per second, the processing time should ensure correct detection of all signals in the spectrum coming from a given radio station. For RFROI-CNN, the processing time depends on the time and frequency resolution (the neural network part) and the number of detected signals in the spectrum (the ROI Segmenter part). Examples of average CNN, ROI-Segmenter processing time and average fps (frames per second) for an example distribution of real FM radio broadcast band spectrum from Figure 11 are given in Table 2. The results were collected for $f_{s}$ = 42 MHz, $f_{0}$ = 100 MHz, $G_{LNA}$ = 8 dB using the SDR USRP-B210 transceiver, manufactured by Ettus Research, TX, USA, without any external bandpass filter. The neural network was processed on an RTX3060 GPU, manufactured by NVIDIA Corporation, CA, USA, while the ROI-Segmenter ran on an i7-11800H CPU, manufactured by Intel Corporation, CA, USA.

As can be seen from Table 2, the lower the frequency resolution, the faster the noise estimator and ROI-Segmenter blocks, and thus the higher the fps obtained. On the other hand, for the same fft resolution value, at low time resolutions (e.g., 32) the CNN processing time is noticeably longer compared to higher values. For example, for fft resolution = 8192 and time resolution = 32 avg, cnn processing time is 21.75 ms, while for time resolution = 128, it is 10.97 ms, almost doubling the processing time with four times less data. This may be due to a significant deviation from the aspect ratio on which the network was trained. However, this does not have a direct impact on the average fps value, because at higher time resolutions, the ROI-Segmenter block based on OpenCV algorithms needs more time to reprocess feature maps into bounding boxes.

The obtained detection time for high resolutions may be a limitation of the presented method in professional applications compared to very fast technical methods based on energy detection and thresholding. However, in its current form, the method has been tested for online detection using an SDR module with good results, indicating that further research aimed at improving the computational performance of this method could provide a valuable contribution to the field of wideband spectrum sensing and can help solve the problem of lack of frequencies in the radio spectrum, as well as allowing for an advantage in jamming and jamming-avoiding in tactical communications, which can be one of the AI applications in military systems [61].

## 6. Conclusions

This paper proposes the RFROI-CNN solution, which is the application of deep convolutional neural network in the detection of RF signals in a broadband spectrum. This method extends energy detection approach by precise estimation of the noise distribution in the spectrogram, enabling the detection of significantly weaker signals than traditional thresholding methods commonly used in wideband spectrum sensing. By utilizing convolutional neural networks to analyze radio spectrograms, the estimation of noise distribution is performed through convolutions with a kernel size of 3 × 3, taking into account neighboring signal power values in the analyzed spectrogram segment. Signal detection is achieved by subtracting the noise estimate from the input spectrogram.

Analysis of radio spectrograms using convolutional neural networks for various modulations, sampling frequencies and observation times confirms the effectiveness of the RFROI-CNN approach, surpassing the popular energy detection method by more than 6 dB. This allows for the detection of signals with low SNR values in the wideband spectrum.

Although the RFROI-CNN solution is trained on synthetic data, it proves to be effective in real radio environments. However, further testing and validation of this approach on different scenarios, real-world datasets and diverse conditions are recommended to better evaluate its performance and practical applicability.

The application of RFROI-CNN in analyzing real radio environments opens up possibilities for specialized devices such as cognitive spectrum analyzers, cognitive radios or intelligent AI-based radio stations. AI-based radio stations can help address the issue of limited frequency availability in the radio spectrum and provide advantages in jamming and jamming-avoidance in tactical communications. This also highlights the potential applications of artificial intelligence in military communication systems.

The direction of future research will focus on the usage of other more optimal neural network architectures’ noise distribution estimation in the spectrogram. Additionally, employing generative models to enhance performance, training networks on real-world wideband spectrum I/Q samples and evaluating the effectiveness and efficiency of this solution in various radio conditions, considering different types of radio signals, interferences and noise, are crucial areas to explore.

Furthermore, exploring the adaptability of this solution to other domains such as medicine or industry, where signal detection in noisy environments is equally important, would be valuable. This would involve investigating the applicability and potential improvements of the RFROI-CNN approach beyond the realm of radio spectrum analysis, addressing challenges in signal detection and noise estimation in diverse fields.

In conclusion, the research findings presented in this article open up new perspectives in RF signal detection in wideband spectrum through deep learning. The RFROI-CNN solution holds practical potential across different fields, and continued research and development can contribute to further improving its effectiveness and efficiency.

## Figures and Tables

**Table 1:** Comparison of mAP results for different modulations and PSNR in the test database. Inbold value indicates better performance.

Wss Method	Test Dataset	mAP (IoU = 0.5) (%)	mAP (IoU = 0.05) (%)
RFROI-CNN	rfspec-db-test(M(AM; FM; CW; LSB; USB), PSNRdB(0; 8))	**41.85**	**57.6**
ED (best λ)	rfspec-db-test(M(AM; FM; CW; LSB; USB), PSNRdB(0; 8))	7.54	16.54
RFROI-CNN	rfspec-db-test(M(AM; FM; CW; LSB; USB), PSNRdB(−8; 8))	**24.44**	**36.62**
ED (best λ)	rfspec-db-test(M(AM; FM; CW; LSB; USB), PSNRdB(−8; 8))	3.74	8.85
RFROI-CNN	rfspec-db-test(M(AM; FM; CW), PSNRdB(0; 8))	**76.9**	**83.67**
ED (best λ)	rfspec-db-test(M(AM; FM; CW), PSNRdB(0; 8))	16.13	32.11
RFROI-CNN	rfspec-db-test(M(AM; FM; CW), PSNRdB(−8; 8))	**55.27**	**69.74**
ED (best λ)	rfspec-db-test(M(AM; FM; CW), PSNRdB(−8; 8))	9.23	20.13
RFROI-CNN	rfspec-db-test(M(LSB; USB), PSNRdB(0; 8))	**3.91**	**22.55**
ED (best λ)	rfspec-db-test(M(LSB; USB), PSNRdB(0; 8))	0.01	1.33
RFROI-CNN	rfspec-db-test(M(LSB; USB), PSNRdB(−8; 8))	**1.73**	**11.32**
ED (best λ)	rfspec-db-test(M(LSB; USB), PSNRdB(−8; 8))	0.01	1.14

**Table 2:** RFROI-CNN performance comparison for different temporal and fft resolutions for GPU (CNN) and CPU (ROI-SEGM).

Fft Resolution	Time Resolution	Avg Cnn Time (ms)	Avg Roi-Segm Time (ms)	Avg Bboxes Count	Avg Fps
8192	32	21.75	65.31	15.00	10.00
8192	64	17.13	143.22	22.30	5.70
8192	96	14.09	182.92	30.00	4.70
8192	128	10.97	254.59	38.60	3.50
4096	32	20.77	27.34	13.70	17.60
4096	64	17.02	63.47	20.30	11.10
4096	96	14.79	87.29	24.20	8.90
4096	128	13.13	123.35	28.10	6.80
2048	32	13.16	11.09	14.00	38.50
2048	64	14.23	30.77	17.90	19.70
2048	96	12.86	40.08	26.70	16.90
2048	128	11.96	56.64	27.10	13.20
1024	32	11.95	6.89	13.90	48.10
1024	64	7.12	14.12	19.90	44.40
1024	96	7.63	17.93	27.50	36.60
1024	128	10.20	24.15	32.80	26.60
512	32	9.26	4.02	12.30	71.50
512	64	9.35	6.61	20.30	57.70
512	96	5.04	10.45	31.40	61.20
512	128	5.41	13.25	36.50	50.60

## Data Availability

The training dataset used to train the neural network was prepared by the authors and publicly shared at: https://github.com/aolesinski/rfspec-db, accessed on 18 May 2023.
